# Metabolite Profiling Analysis of the Tongmai Sini Decoction in Rats after Oral Administration through UHPLC-Q-Exactive-MS/MS

**DOI:** 10.3390/metabo14060333

**Published:** 2024-06-14

**Authors:** Xianhui Zheng, Yingying Zhan, Mengling Peng, Wen Xu, Guanghai Deng

**Affiliations:** 1 The Second Clinical College, Guangzhou University of Chinese Medicine, Guangzhou 510006, China; xianhuizheng@gzucm.edu.cn (X.Z.); 20221110784@stu.gzucm.edu.cn (Y.Z.); 20221110783@stu.gzucm.edu.cn (M.P.); fantacy@gzucm.edu.cn (W.X.); 2 Guangdong Provincial Hospital of Chinese Medicine, Guangzhou 510120, China

**Keywords:** Tongmai Sini decoction, metabolites profiles, metabolic pathways, UHPLC-Q-Exactive-MS/MS

## Abstract

Tongmai Sini decoction (TSD), the classical prescriptions of traditional Chinese medicine, consisting of three commonly used herbal medicines, has been widely applied for the treatment of myocardial infarction and heart failure. However, the absorbed components and their metabolism in vivo of TSD still remain unknown. In this study, a reliable and effective method using ultra-performance liquid chromatography coupled with hybrid quadrupole-Orbitrap mass spectrometry (UHPLC-Q-Exactive-MS/MS) was employed to identify prototype components and metabolites in vivo (rat plasma and urine). Combined with mass defect filtering (MDF), dynamic background subtraction (DBS), and neutral loss filtering (NLF) data-mining tools, a total of thirty-two major compounds were selected and investigated for their metabolism in vivo. As a result, a total of 82 prototype compounds were identified or tentatively characterized in vivo, including 41 alkaloids, 35 phenolic compounds, 6 saponins. Meanwhile, A total of 65 metabolites (40 alkaloids and 25 phenolic compounds) were tentatively identified. The metabolic reactions were mainly hydrogenation, demethylation, hydroxylation, hydration, methylation, deoxylation, and sulfation. These findings will be beneficial for an in-depth understanding of the pharmacological mechanism and pharmacodynamic substance basis of TSD.

## 1. Introduction

The classical prescriptions of traditional Chinese medicine (TCM) have originated from the fixed combination of certain kinds of herbal medicines recorded in the ancient classics, which have been still widely used in East Asia and exhibited precise clinical efficacy, with obvious characteristics and advantages [1]. For a long time, some classic prescriptions have been developed into modern Chinese medicinal preparations through the optimization of preparation technology and drug development research [2,3]. Most classical prescriptions have existed for at least hundreds of years, and with time, classic prescriptions may have changed to some extent, while their core characteristics (e.g., composition of herbs, proportion of herbs, etc.) have not changed significantly [4,5]. The reasons for the inheritance of classical prescriptions to the present day can be attributed to the high safety of the prescriptions and their proven efficacy due to a large number of clinical applications.

Tongmai Sini decoction (TSD) is a classic formula from the Chinese medical masterpiece “The Treatise on Typhoid Fever”, written 1800 years ago. It consists of three herbal concoctions of *Radix Aconiti Lateralis Preparata* (RALP), *Rhizoma Zingiberis* (RZ), and *Radix Glycyrrhizae Preparata* (RGP) and is commonly used in modern times for myocardial infarction and heart failure, atherosclerosis, shock, diarrhea, etc. [6,7]. TSD has the effects of raising blood pressure, strengthening the heart, anti-hypoxia, anti-shock, anti-thrombosis, anti-myocardial ischemia, anti-slowing arrhythmia, and so on [8,9]. The main chemical constituents of TSD include alkaloids (from RALP), phenolic acids and saponins (from RGP), and volatile oils (from RZ). At present, chemical composition [10], pharmacological, pharmacokinetic [11,12,13], and metabolomics [8,9,14] studies have been preliminarily conducted on TSD, especially on its cardiovascular activities. Most of the studies on TDS concentrated on the pharmacokinetics of diterpene alkaloids after oral administration of TDS, and some of the studies focused on the changes in the in vivo metabolome or lipidome profile against myocardial ischemia, heart failure, hypothyroidism. There is a lack of systematic and in-depth in vivo chemical and metabolite studies of TDS.

The components of different botanicals enter the body and produce metabolites, which exert therapeutic effects through multiple pathways [15]. To fully understand the therapeutic components, it is necessary to first analyze the blood-entering components and their metabolites, as well as to study the metabolites distributed in plasma, urine, feces, and tissues, which is conducive to analysis of the potential components and pathways of action in the body [16,17].

High-resolution mass spectrometry (HRMS), in combination with chromatography technology, has provided useful structural information about chemical components, offering strong support for the characterization of in vivo and in vitro metabolic components of botanicals [18,19,20]. In recent years, in order to improve the sensitivity and selectivity of obtaining MS/MS or MS^n^ data for trace components in vivo, some acquisition and identification strategies have been developed, combined, and applied, for instance, the extracted ion chromatogram (EIC), mass defect filter (MDF), dynamic background subtraction (DBS) and neutral loss filter (NLF) [21,22,23].

In this paper, the established UHPLC-Q-Exactive-MS/MS methods have great advantages for the qualitative analysis of bioactive samples in rats after oral doses of TSD, and a variety of post-data processing techniques, including EIC, MDF, DBS, and NLF was applied for quickly screen and systematically identify the metabolites. These metabolic studies can provide the chemical foundation and an in-depth understanding of metabolic transformation for further research on effective substances and the action mechanism of TSD.

## 2. Materials and Methods

### 2.1. Chemicals and Plant Materials

Aconitine, mesaconitine, hypaconitine, liquiritin, liquiritigenin, ononin, and formononetin, with purities greater than 98%, were purchased from Shanghai Yuanye Biological Technology Co., Ltd. (Shanghai, China). HPLC-grade acetonitrile, methanol, and formic acid were purchased from Sigma Aldrich (St. Louis, MO, USA). Ultrapure Water (18.2 MΏ) was produced by a Milli-Q water system (Millipore, Bedford, MA, USA). The herbal pieces of RALP, RZ, and RGP were purchased from Kangmei Pharmaceutical Co. Ltd. Of Guangdong, China, and identified by Prof. Zhi-hai Huang, The Second Clinical College, Guangzhou University of Chinese Medicine, Guangzhou, China.

### 2.2. Plant Extract Preparation

According to the documentary records of TSD, the TSD pieces that included RALP (30 g), RZ (20 g), and RGP (30 g) were soaked with 8 times the amount of water for 30 min and decocted to boil (100 °C) for 2 h. The filtrate was collected, and the residue was decocted in 8 times the amount of water for 1.5 h again. The hot filtrate was combined and concentrated to 80 mL (1 g herbal pieces/1 mL aqueous solution). The obtained TSD extract was stored at −20 °C before use.

### 2.3. Animal and Drug Administration

Male Sprague–Dawley rats (220–260 g) were obtained from Guangdong Provincial Medical Laboratory Animal Center (Guangdong, China). All animal experiments were performed at the SPF animal laboratory [experimental animals license number SYXK (Guangdong, China) 2008–0094]. The Institutional Animal Ethics Committee of Guangdong Provincial Hospital of Chinese Medicine approved all experimental protocols (No. 2023131).

Six SD rats were randomly divided into two groups (Urine and plasma groups) and adapted to the metabolic cage for a week before the experiment. Blank urine and plasma samples were collected under abrosia state ahead of gastric gavage. The rats were fasted for 14 h with water ad libitum before oral administration of TSD extract and underwent 4 h of water deprivation after that. TSD extract was orally administered to rats of urine and plasma groups twice at an interval of 1 h, and the dosage was 2 mL per 100 g bodyweight per time.

### 2.4. Sample Collection and Pretreatment

Urine samples from 0 to 24 h after the second dosing were collected and stored at −80 °C prior to analysis. Plasma samples were obtained at 1, 2, 4, 8, and 12 h after the second administration in heparinized 1.5 mL polythene tubes under diethyl ether anesthesia, respectively. All plasma samples were centrifuged at 4000 rpm for 10 min, and the plasma supernatants were then merged in equal volume and frozen at −80 °C prior to analysis.

The collected urine and plasma samples (200 μL) were added with 4× the volume of acetonitrile-methanol (3:1) to precipitate protein, respectively. All separate supernatants were dried under N2 flow, and the residues were resuspended in 200 μL acetonitrile and centrifuged at 15,000× *g* for 8 min. Finally, a 5 μL sample was injected into the UHPLC-Q-Exactive-Orbitrap MS system for further analysis.

### 2.5. Instrumentation and Conditions

LC analyses were conducted on a Thermo UltiMate 3000 UHPLC system (Thermo Fisher Scientific, San Jose, CA, USA) equipped with a quaternary pump, a cooling autosampler, and a thermostatically controlled column oven. An ACQUITY UPLC HSS T3 Column (2.1 × 100 mm, 1.8 μm) was used. The mobile solvents were composed of acetonitrile (A) and water with 0.02% formic acid (B), and the gradient elution profile was employed as follows: 5% A, 0 min; 16% A, 12 min; 55% A, 23 min; 90% A, 35 min; 95% A, 40 min; returning to initial conditions in 4 min at a flow rate of 200 μL/min at room temperature. The injection volume was 5 μL. The temperatures of the sample tray and the column oven were set at 4 and 35 °C, respectively.

A Q-Exactive hybrid quadrupole-orbitrap mass spectrometer was connected to an LC system via an electrospray ionization source as an interface. Data acquisition and processing were calculated using Compound Discoverer 3.2 software. The optimized parameters for MS analysis were as follows: the mass spectrometer parameters were positive (PI) and negative (NI) ion mode; the resolution of the Orbitrap mass analyzer was set as 30,000; ion spray voltage was −3.8 kV; the capillary temperature was 325 °C; the sheath gas flow rate was 40 psi; the auxiliary gas flow rate was 8 psi; and the mass range was *m*/*z* 150–1500. The properties of data-dependent MS^2^ scanning (DDS) parameters and events were as follows: resolution, 17,500; HCD, 35 eV; repeat count, 2; exclusion list, 50; repeat duration, 5 s; and exclusion duration, 30 s. The mass error for molecular ions of all compounds identified was within ±5 ppm.

## 3. Results and Discussion

### 3.1. Systematic Analytical Strategy for Online Metabolite Analysis

**Figure 1:**
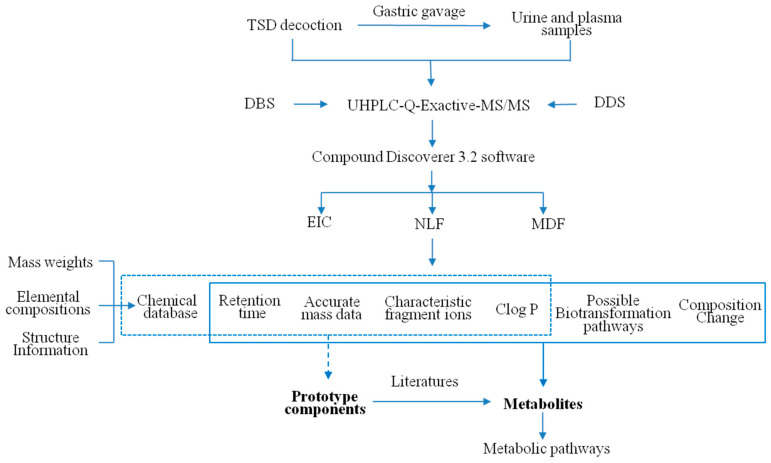
Workflow of the analytic strategy for the metabolite identification of TSD.

Based on our previous research on the cleavage patterns of components in RALP and RGP and a review of the literature [24,25,26,27,28], the metabolite profiling of TSD was systematically investigated by UHPLC-Q-Exactive-MS/MS methods. The workflow of the analytic procedure was carried out and shown in Figure 1. Figures S1 and S2 (Supplementary Materials) displayed the detailed workflow for the identification of prototype components and metabolites, respectively.

The strategy consisted of the following steps: (1) First, the chemical database (Table S1, Supplementary Materials) was constructed, including mass weights, elemental compositions, and structure information of chemical compositions originating from RALP, RGP, and RZ based on our previous research and the related literature [24,25,26,27,28,29]. (2) Then, an online full-scan and MS/MS data acquisition was processed in both negative and positive modes based on the DBS and DDS techniques for potential metabolite detection. (3) Next, the data files were imported into the Compound Discoverer 3.2 software, and the data-mining tools of EIC, NLF, and MDF were applied to screen the possible metabolites of TSD. Table S2 (Supplementary Materials) showed the detailed parameters of data processing. The main compounds with mass spectral peak areas greater than 10^8^ in the decoction (shown in Table 1) were used as parent compound templates for MDF data screening (±50 mDa) (4) Next, based on the chemical database, acquired accurate mass data, retention time, and characteristic fragment ions, the identification of prototype components was elucidated (shown in Table 2). In addition, the Clog *p* values calculated by ChemDraw 14.0 were used to distinguish isomers at different retention times. (5) Finally, the mass information of potential metabolites, as well as their possible biotransformation pathways and composition change given by Compound Discoverer 3.2, were compared by the data of prototype components and the related literature to verify the metabolites and their metabolic pathways (shown in Table 3).

### 3.2. Identification of Prototype Components

**Figure 2:**
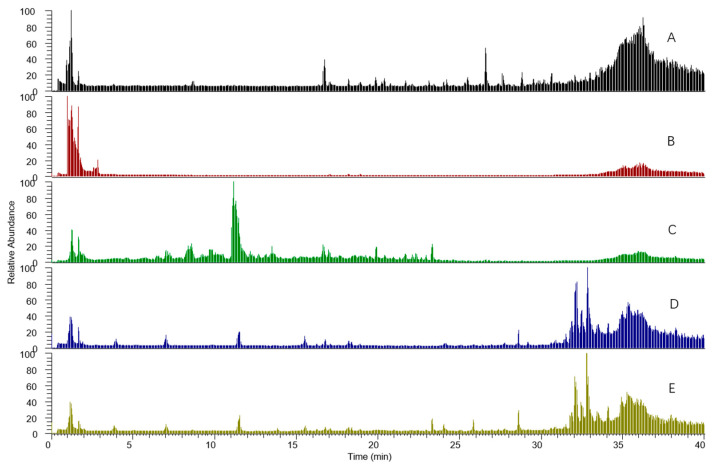
Total ion chromatograms (TIC) of TSD and the urine and plasma samples after oral administration by UHPLC-Q-Exactive-MS/MS ((**A**): Tongmai Sini decoction; (**B**): blank urine samples; (**C**): urine samples; (**D**): blank plasma samples; (**E**): plasma samples.).

An in-house database has been established for each compound involved in RALP, RGP, and RZ based on our previous experimental data and the related literature for the investigation of their chemical constituents. The database consisted of the compound name, molecular formula, accurate molecular mass, chemical structure, MS^2^ mass spectra, and related product ion information. The total ion chromatograms (BPIs) of TSD and the urine and plasma samples after oral administration by UHPLC-Q-Exactive-MS/MS in positive and negative ion modes are presented in Figure 2. It is found that the majority of alkaloids responded well in the positive mode, and the majority of phenolic compounds and saponins responded well in the negative mode. A total of 82 prototype compounds were identified or tentatively characterized, including 41 alkaloids, 35 phenolic compounds, and 6 saponins (shown in Table 2) by comparing the EICs among TSD, drugged, and blank samples and by comparison with reference standards, internal database, and the literature. Figure S3 (Supplementary Materials) displayed MS/MS spectra of major prototype compounds in the urine samples.

#### 3.2.1. Identification of Alkaloid Components

Metabolites for alkaloids obtained in this study could be classified into three subtypes, namely, diester-diterpenoid alkaloids (DDAs), monoester-diterpenoid alkaloids (MDAs), and amine-diterpenoid alkaloids (ADAs) [30]. We conducted an in-depth study of the chemical constituents of alkaloids of *Aconitum carmichaeli* in previous research [24,29], in which we carried out detailed mass fragmentation analysis of DDAs, MDAs, and ADAs, and a total of 42 DDAs and 120 diterpenoid alkaloids were identified, respectively.

In the MS^2^ spectra of DDAs, the most abundant ion yielded from the loss of a molecule of AcOH at the C_8_ site, which could be a diagnostic neutral loss for the differentiation of DDAs from MDAs and ADAs [29]. Thus, Compounds **29**, **33**, **35–38,** and **40–41** were extracted by NLF for 60 Da in MS spectra for the urine sample, showing their molecular weight between 600 and 650 Da. Among them, Compounds **33**, **36**, and **37** were unambiguously identified as mesaconitine (MA), aconitine (AC), and hypaconitine (HA), respectively, by comparing their t_R_ values and mass spectra data with those of reference compounds. Apart from the ion of [M+H-60(AcOH)]^+^ (*m*/*z* 572.2844, 586.3002, 556.2903), the ions of [M+H-60-32(MeOH)]^+^ (*m*/*z* 554.2727, 524.2634, 540.2551) and [M+H-60-32(MeOH)-28(carbonyl group)]+ (*m*/*z* 526.2797, 496.2750, 522.2487) of the three compounds, respectively, suggested the active elimination of MeOH occurred at C16 site and a neutral molecule of CO, which could also be regarded as characteristic fragments for identification of the DDAs. Compounds **29**, **35**, **38,** and **40–41** were tentatively identified as 10-OH-mesaconitine, dehydrohypaconitine, secoyunaconitine, 3-deoxyaconitine, and chasmaconitine by comparing their acquired accurate mass data, characteristic fragment ions with those of compounds in our previous research [29].

In the MS spectra for the urine sample, by extraction of NLF for both 32 Da and 18 Da with limitation of molecular weight ranging from 500 to 620 Da, ten peaks were found. Neutral losses of 32, 18, and 122 Da, corresponding to the elimination of acetic acid, methanol, and benzoic acid, or combinations of these, could be considered diagnostic fragment ions for MDAs [31]. However, fragment peaks formed by the loss of the typical substituent group as BzOH (122 Da) were hardly detected for MDAs in this study. Thus, Compounds **22–28** and **30–32** were identified as MDAs accordingly by comparing the accurate mass data and diagnostic fragment ions with those of the compounds in our previous research [24].

**Figure 3:**
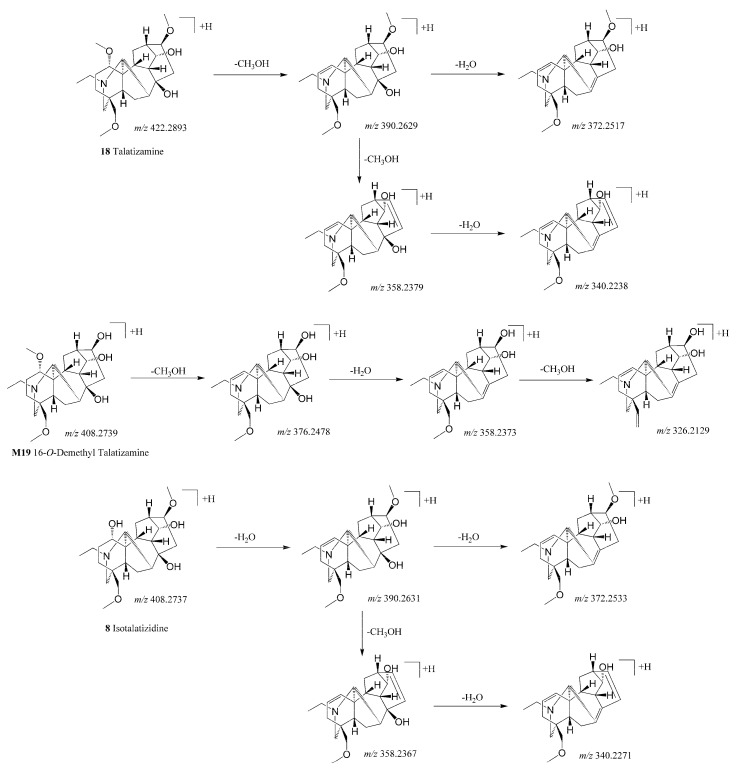
Probable fragmentation pathways of talatizamine, isotalatizidine, and 16-*O*-demethyl talatizamine.

A total of 21 prototype compounds were identified as ADAs, most of which possessed molecular weight between 390 and 500 Da and were eluted within the initial 16 min. The substitutions of C_1_ and C_3_ sites of ADAs were relatively active sites and could be easily cleaved, yielding major peaks [M+H-H_2_O]^+^ or [M+H-CH_3_OH]^+^ in MS^2^ spectra as the diagnostic ion accordingly. Fragmentation pathways of differently substituted ADAs included different diagnostic ions. Compounds **1**, **4**, **6**, **7**, **8**, **13**, **14**, as ADAs with C_1_-OH substitution, firstly fragmented into [M+H-H_2_O]^+^ as diagnostic fragment ions and followed by losses of typical substituent groups (CH_3_OH and H_2_O) in their MS^2^ spectra. By comparing their accurate mass data with our chemical database and the literature [24,32], they were identified as karakolidine, senbusine A, senbusine B, karakoline, isotalatizidine, fuziline, and neoline, respectively. For Compounds **18** (talatizamine), the most prominent fragmentation ions were designated as 390.2696 ([M+H-CH_3_OH]^+^), suggesting its C_1_ site with -OCH_3_ substitutions. It also yielded 372.2517 ([M+H-CH_3_OH-H_2_O]^+^), 358.2379 ([M+H-CH_3_OH-CH_3_OH]^+^), and 340.2238 ([M+H-CH_3_OH-CH_3_OH-H_2_O]^+^), and its characteristic fragmentation patterns are shown in Figure 3.

#### 3.2.2. Identification of Phenolic Compounds

In addition to alkaloids from RALP, the main prototype compounds identified in vivo included flavonoids, isoflavonoids, coumarins, and saponins from RGP, and volatile oils from RZ, as shown in Table 2. The MS data of these compounds were compared with those of reference standards, internal databases, and the literature, while isomers could be initially identified by comparing their ClogP.

Flavonoids are important active components of RGP, among which four components, namely liquiritigenin, isoliquiritigenin, iquiritin, and isoliquiritin, have the highest content and are regarded as the indicator components of RGP, which were identified by comparing mass data with those of the reference standards. Compound **47**, as reference compound liquiritin, formed the [M-H]^−^-based peak at *m*/*z* 417.11890 (C_21_H_21_O_9_^−^), for which furtherly formed fragmentation ion *m*/*z* 255.0662 [M-H-glu]^−^ of the aglycone element in the MS/MS spectrum, accompanied by three characteristic fragments at *m*/*z* 135.0074 (C_7_H_3_O_3_^−^), 119.0488 (C_8_H_7_O^−^), and 91.0173 (C_6_H_3_O^−^), which can be used for the identification of the same type of licorice flavonoids.

Compound **50** formed the [M+H]^+^ molecular ion peak at *m*/*z* 431.13280 and further removed one molecule of glucose residues to form the aglycone at *m*/*z* 269.08121, which was identified as ononin, the main isoflavone of RGP. Its aglycone formed the same ion at *m*/*z* 269.08170 at the retention time of 26.58 min and was fragmented into the fragments of *m*/*z* 253.0497, 237.0554, and 213.0911, which is identified as formononetin, and the two prototypes are the most important isoflavonoid components in RGP.

The elemental compositions of other types of licorice flavonoid constituents determined by LC-MS were compared with the data of existing database compounds. Compounds **44**, **53**, **54**, **67,** and **68** were preliminarily identified as 5-hydroxyliquiritin, licochalcone B, dihydroxyflavone, licoflavone A, and isolicoflanonol. Similarly, other types of phenolic compounds, such as coumarins, were identified or preliminarily identified, including Compounds **60**, **66**, **64**, **70**, **73,** and **74**, which were identified as glycycoum-arim, licocoumarione, licopyranocoumarin, glycyrin isoglycyrol, and glycyrol, correspondingly. A few other phenolic components observed in vivo of TSD were derived from RZ, while compounds **56**, **63**, and **75** were tentatively identified as 6-gingerol, 6-shogaol, and 10-shogaol, respectively, with fragment ions *m*/*z* 177.09 and 137.06 as their characteristic fragment ions in PI mode, which is consistent with the literature [33].

#### 3.2.3. Identification of Saponins

From the LC-MS/MS profiles, six saponin components were found as absorbed prototype components, all of which were derived from RGP. The saponins (Compounds **77**, **78**, **79**, **81,** and **82**) were within the retention time of 14–21 min and had both mass spectral response in NI and in PI mode.

As a general rule for triterpenoid saponins in MS/MS spectra, the fragmentation reactions undergone by activated saponin ions almost occur within the glycan part of the saponin ions, and the sugar chains can be eliminated successively from end to inner and finally to obtain an aglycone ion [34]. Through glycosidic cleavages or cross-ring cleavages, the parent ion obtained a series of ions retaining the charge at the reducing terminus were termed Y and Z (glycosidic cleavages) and X (cross-ring cleavages), whereas those ions retaining the charge at the non-reducing terminus are termed B, C (glycoside cleavages), and A (cross-ring cleavages) [35].

The MS cleavage pathways of saponins from RGP, however, were incompletely abided by this rule. Take glycyrrhizic acid as an example; in MS spectra of PI mode, the ions of [M-H]^−^ were obtained, accompanied by the fragment ions of *m*/*z* 647.37744 [M+H-β-D-glucuronopyronosyl (glcA)]^+^ and *m*/*z* 453.33554 [aglycottne (agl)+H-H_2_O]^+^, which were similarly for the other detected saponins and has not been reported up to present. More interestingly, in the MS/MS spectra of the detected saponins, the ions of [agl+H-H_2_O]^+^ rather than [agl+H]^+^ were observed as the base peaks, namely, *m*/*z* 453.34 (C_30_H_45_O_3_^+^), 469.33 (C_30_H_45_O_4_^+^), and 511.34 (C_30_H_45_O_4_^+^), corresponding to the aglycone of enoxolone, hydroxyenoxolone, and acetoxyenoxolone, respectively.

The produced ions obtained in NI mode were quite different from those in PI mode. The fragment ions of glycosidic cleavages or cross-ring cleavages, as well as the aglycone, were hardly detected in NI mode. The ions of *m*/*z* 351.05 (C_12_H_15_O_12_^−^), 193.03 (C_6_H_9_O_7_^−^), 175.02 (C_6_H_7_O_6_^−^), and 113.02 (C_5_H_5_O_3_^−^) were observed, corresponding to the successive loss of two glucuronopyranosyls. Thus, the identification information for aglycone s and sugar chains of licorice saponins can be obtained from PI and NI ion modes, respectively.

### 3.3. Identification of Metabolites

Prototypes and metabolites exist simultaneously in plasma and urine samples. Thirty-two major prototypes, including 11 alkaloids from RALP, as well as 21 phenolic and saponin compounds from RGP and RZ, were selected as MDF templates for metabolite screening. The 32 compounds contained a wide range of chemical structure types with relatively high content in TDS. A total of 40 alkaloids and 25 phenolic compounds were identified or tentatively characterized by comparing the mass data with those of prototype compounds and metabolic pathways reported by the literature [36,37,38,39,40].

After prototypes are absorbed into the body, some of them are excreted as prototypes, and some of them can be converted into other metabolites. DDAs were ester hydrolyzed to MDAs in rats; for example, MA, HA, and AC could be ester hydrolyzed to 14-Benzoylmesaconine (BM), 14-Benzoylhypaconine (BH), and 14-Benzoylaconitine (BA) during the process of metabolism in rat, while BM, BH, and BA themselves could be metabolized to mesaconine, hypaconine, and aconine [36]. Therefore, certain prototypes are themselves metabolites and metabolized from other prototypes in rats.

#### 3.3.1. Identification of Alkaloid Metabolites

For diterpenoid alkaloids, most metabolites from hydroxylation, deoxylation, demethylation, deethylation, dehydrogenation, ester hydrolysis, and demethylation with deoxylation have been found in vivo. Metabolites of alkaloids were identified or tentatively identified based on their metabolic pathways, as reported in the literature [37].

The metabolites for major alkaloids were found in the urine and plasma samples, as displayed in Table 3. Most metabolites observed were mainly metabolized from karakolidine, songorine, karakoline, talatizamine, hypaconitine, mesaconitine, neoline, and fuziline. These results manifested that alkaloids mainly underwent oxidation, dehydrogenation, demethylation, N-deethylation, hydrolysis, demethylation with deoxidation, and dehydrogenation with demethylation, etc.

After oral administration of TSD, eight related metabolites of talatizamine (**18**) were identified in urine samples. Metabolite **M18** and **M19** showed [M+H]^+^ ion at *m/z* 408.27386 and 408.27393 (giving formula C_23_H_37_NO_5_), 14 Da (CH2) less than the parent compound. In the MS^2^ spectra, characteristic ions at *m*/*z* 376.25 ([M+H-CH_3_OH]^+^), 358.24 ([M+H-CH_3_OH-H_2_O]^+^), and 326.21 ([M+H-CH_3_OH-H_2_O-CH_3_OH]^+^), suggesting its C_1_ site with -OCH_3_ substitutions. Those characteristic ions were different from the characteristic ions of the prototype component, isotalatizidine (Compound **8**), although they shared the same elemental composition (C_23_H_37_NO_5_). Isotalatizidine, with -OH substitutions at the C_1_ site, first yielded 390.2631 ([M+H-H_2_O]^+^) by loss of H_2_O at the C_1_ site. The fragmentation pathways of demethyl talatizamine and isotalatizidine can be compared in Figure 3. The methyl group of the C_16_ site or C_18_ site could easily be metabolized instead of that of the C_1_ site for **M18** and **M19.** The Clog *p* values of 18-*O*-demethyl talatizamine and 16-*O*-demethyl talatizamine were −0.78 and −0.74, calculated by ChemDraw 14.0. Hence, **M18** and **M19** were tentatively determined as 18-*O*-demethyl talatizamine and 16-*O*-demethyl talatizamine.

**M4** was confirmed as hydroxylated talatizamine for the [M+H]^+^ ion at *m*/*z* 438.28433 (formula C_24_H_39_NO_6_), 16 Da (O) more than talatizamine, and the fragment ions at *m*/*z* 406.2588, 388.2476, 374.230 and 356.2226 were all 16 Da less than those of talatizamine. Therefore, **M4** was deduced as 10-Hydroxy Talatizamine, as for the C_10_ site in diterpenoid alkaloids prone to be hydroxylated by the literature [38].

**Figure 4:**
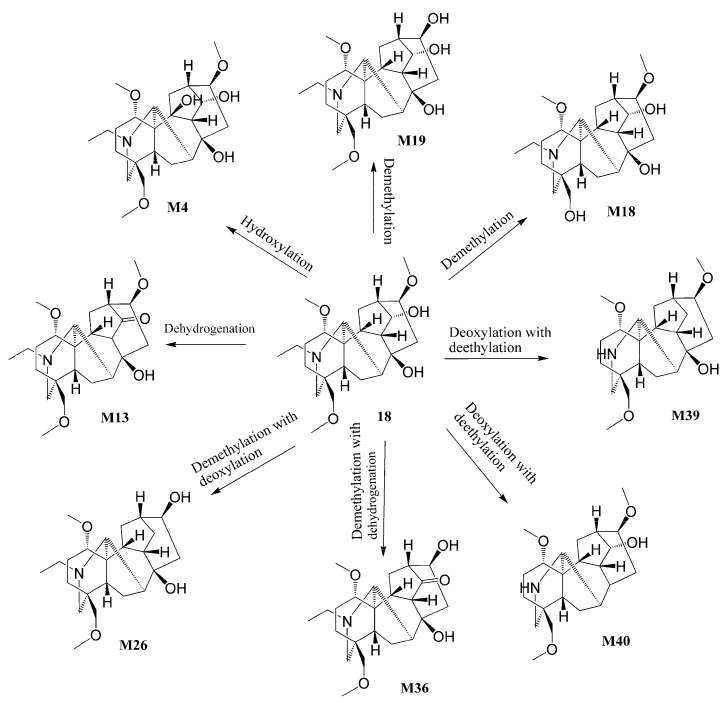
Proposed metabolic pathways of talatizamine in vivo.

Apart from these three metabolites, other metabolites (**M13**, **M26**, **M36**, **M39,** and **M40**) of talatizamine were produced through the reaction of dehydrogenation, demethylation, N-deethylation, and deoxidation. The proposed metabolic pathways of talatizamine are shown in Figure 4. The other metabolites of alkaloids were deduced accordingly by their acquired accurate mass data, retention time, and characteristic fragment ions, as well as the Clog *p* values, and biotransformation pathways information and composition change calculated by ChemDraw 14.0 and Compound Discoverer 3.2.

#### 3.3.2. Identification of Phenolic Compound Metabolites

Metabolites of phenolic compounds, mainly from hydroxylation, oxylation, methylation, dehydrogenation, hydration, methylation with oxylation, dehydrogenation with oxylation, and sulfation, have been observed in vivo. They were identified or tentatively identified by comparing their accurate mass data with prototypes and their metabolic pathways reported by the literature [39,40]. The metabolites for major phenolic compounds found in the urine and plasma samples were exhibited in Table 4.

Metabolites of phenolic compounds observed in vivo were mainly derived from the metabolism of liquiritigenin, isoliquiritigenin, and 6-gingerol, which were the most important aglycones from RGP and RZ in TSD. According to the MS data and the metabolic pathways reported in the literature, eleven related metabolites were identified in urine and plasma samples after the absorption of liquiritigenin and isoliquiritigenin. **M60** and **M61** showed [M-H]^−^ ion at *m*/*z* 285.0765 (C_16_H_13_O_5_^+^), 30 Da (CH2+O) heavier than parent compounds. In the MS/MS spectra, characteristic ions at *m*/*z* 270.05 [M-H-CH_2_]^−^ indicated the methyl substitution, and *m*/*z* 135.01 or 119.05 were used for the characterization of liquiritigenin or isoliquiritigenin derivatives. According to the polarity of liquiritigenin and isoliquiritigenin, **M60** and **M61** were confirmed as methyl-hydroxy liquiritigenin and methyl-hydroxy isoliquiritigenin. In vivo, liquiritigenin and isoliquiritigenin could be metabolized to a series of metabolites (**M41**, **M42**, **M50**, **M53**, **M54**, **M56**, **M57**, **M60**, **M61**, **M63,** and **M64**) by reaction of hydrogenation, dehydrogenation, hydroxylation, oxylation, hydration, methylation, and sulfation. In vivo, seven metabolites (**M43**, **M47**, **M48**, **M52**, **M55**, **M58,** and **M59**) of 6-gingerol were produced through the reaction of hydrogenation, methylation, hydration, hydroxylation, and dehydrogenation, with characteristic ions at *m*/*z* 163.08 or 137.06.

### 3.4. Difference between Urine and Plasma Samples

Xenobiotics usually vary at trace levels and are interfered with endogenous components. Comparative analysis of metabolites between plasma and urine samples was carried out by the same LC-MS/MS method. Most prototype components and metabolites possessed suitable signal responses in urine samples, mainly as metabolites from phase I metabolism referring to dehydrogenation, demethylation, hydroxylation, deoxylation, and deethylation. A few phase II metabolites were detected in the urine, including sulfate conjugates of liquiritigenin, isoliquiritigenin, and formononetin.

Metabolites of TSD detected in the plasma samples are fewer than those in the urine samples. As for plasma samples, 10 prototype components (eight phenolic compounds and two alkaloids) were detected and tentatively identified, most of which were flavonoid aglycones. Fifteen metabolites derived from neoline, talatizamine, karakoline songorine, and fuziline, as well as sixteen metabolites derived from liquiritigenin, isoliquiritigenin, formononetin, gancaonin M, and 6-gingerol, respectively, were found in plasma samples, which indicated there were fewer metabolites identified in plasma samples. These results are reasonable due to their relatively lower concentration and higher matrix interference in plasma than in urine samples.

In the present study, ADAs and their metabolites from RALP were mainly detected in rats after oral administration of TSD. DDAs are the most toxic but chemically unstable alkaloids in RALP, and the alkaloidal composition changed during concocting and decocting, with DDAs changing to MDAs, and both transformed further to ADAs while the toxicity gradually diminished. ADAs, such as fuziline and neoline, showed activity against pentobarbital sodium-induced cardiomyocyte damage by obviously recovering beating rhythm and increasing the cell viability [41]. Mesaconine and hypaconine showed strong cardiac actions on the isolated perfused bullfrog heart. Moreover, mesaconine has protective effects, including improved inotropic effect and left ventricular diastolic function, on myocardial ischemia-reperfusion injury in rats [42].

Metabolites of licorice flavonoids and 6-gingerol were also mainly detected. Liquiritigenin offers cytoprotective effects against various cardiac injuries, and it could protect against myocardial ischemic injury by antioxidation, antiapoptosis, counteraction mitochondrial dysfunction, and damping intracellular Ca^2+^ [43]. 6-Gingerol was identified as a novel angiotensin II type 1 receptor antagonist for cardiovascular disease by high-throughput screening, which partially clarified the mechanism of ginger regulating blood pressure and strengthening the heart [44]. 6-gingerol administration protected I/R-induced cardiomyocyte apoptosis via the JNK/NF-κB pathway in the regulation of HMGB2 [45].

The results of the in vivo metabolite study of TSD in this study suggested that in the following pharmacokinetic, pharmacological, and efficacy studies, attention should be paid primarily to the ADAs alkaloids, licorice flavonoids, gingerol-6, and their metabolites

## 4. Conclusions

A total of 82 compounds, including 41 alkaloids, 35 phenolic compounds, and 6 saponins, were identified or tentatively characterized in TSD by UHPLC-Q-Exactive-MS/MS. Among them, 32 representative compounds with relatively high mass spectral peak areas and different core structures were selected as parent compound templates for further investigation of their metabolic profiles in rats. In total, 65 metabolites were screened out and tentatively characterized in rats’ urine and plasma based on their MS characteristic fragmentation patterns and information. The main metabolic reactions involved hydrogenation, demethylation, hydroxylation, hydration, methylation, deoxylation, and sulfation. This is a systematic study of in vivo metabolism of TSD, and it will be beneficial for further understanding of the pharmacological and pharmacokinetic study of TSD.

## Figures and Tables

**Table 1:** Main prototype components as parent compound templates for MDF data screening. (mass spectral peak areas greater than 10^8^ in the decoction).

Alkaloids (from RALP)	Phenolic and Saponin Compounds (from RGP and RZ)	
Karakolidine	Liquiritigenin	Formononetin
Fuziline	Isoliquiritigenin	Ononin
Neoline	Liquiritin	Glycyrrhizic Acid
Songorine	Licochalcone B	Glycyrrhetinic Acid
14-Benzoylhypaconine	Licochalcone C	Uralsaponin C
Talatizamine	Licochalcone D	Licoricesaponin G2
Karakoline	Licoflavone C	Glycycoum-Arim
14-Benzoylmesaconine	Licoflavone A	Glycyrol
Mesaconitine	Licoricidin	Glycyrin
Hypaconitine	Licoleafol	6-Gingerol
Aconitine	Gancaonin M	

**Table 2:** Prototype compounds identified or tentatively characterized in the urine and plasma samples after oral administration of TSD.

ID	[M+H]^+^(*m/z*)	Formula	t_R_ (min)	Error (ppm)	ms/ms	Identification	ClogP	Area	
								Urine Plasma	
	Alkaloids								
1.	394.25839	C_22_H_35_NO_5_	3.51	−1.58	376.2474, 358.2376, 344.2229, 326.2116, 243.2516	Karakolidine		+++	
2.	394.25820	C_22_H_35_NO_5_	4.02	−1.42	376.2476, 358.2367, 340.2268, 328.2260, 307.4473, 218.6333	Chuanfumine		+++	
3.	439.25229^[M-H]−^	C_23_H_37_NO_7_	4.25	0.56	392.2438, 344.2226, 295.8235, 193.8604, 146.9375	9-Hydroxysenbusine A		+	
4.	424.26871	C_23_H_37_NO_6_	6.69	−1.55	406.2584, 388.2478, 356.2207, 154.1227	Senbusine A	−2.70	+++	
5.	486.26941	C_24_H_39_NO_9_	7.01	−0.72	454.2438, 436.2322, 404.2069, 378.1887, 372.1793, 319.9836	Mesaconine		++	
6.	424.26871	C_23_H_37_NO_6_	7.74	−1.55	406.2581, 388.2472, 356.2210, 154.1231	Senbusine B	0.16	++	
7.	378.26306	C_22_H_35_NO_4_	8.20	−2.18	360.2524, 342.2431, 328.2268, 242.3140	Karakoline		++	
8.	408.27371	C_23_H_37_NO_5_	8.26	−1.81	390.2631, 372.2533, 358.2367, 340.2271	Isotalatizidine		++	
9.	358.23691	C_22_H_31_NO_3_	9.17	−2.13	340.2265	Songorine		+	
10.	360.25293	C_22_H_33_NO_3_	9.18	−1.09	-	Napelline		++	
11.	330.20569	C_20_H_27_NO_3_	9.70	−2.07	236.8785, 170.7432, 152.4712	Hetisine		++	
12.	470.27435	C_24_H_39_NO_8_	10.51	−1.05	-	Hypaconine		++	
13.	454.27933	C_24_H_39_NO_7_	11.33	−1.26	436.2685, 418.2609, 404.2422, 154.1227	Fuziline		+++	
14.	438.28445	C_24_H_39_NO_6_	11.58	−1.28	420.2736, 402.2617, 388.2472, 356.2214, 278.6899	Neoline		+++	
15.	420.27390	C_24_H_37_NO_5_	12.31	−1.32	402.2632, 384.2512, 370.2359, 342.2414, 324.2322, 251.1396	14-Acetylkarakoline		+	
16.	484.28937	C_25_H_41_NO_8_	12.41	−2.33	-	Deoxyaconine		+	
17.	342.16931	C_20_H_23_NO_4_	12.89	−1.96	297.1120, 282.0887, 237.0910, 219.0804, 191.0860	N-Methyl-laurotetanine		+++	
18.	422.28931	C_24_H_39_NO_5_	13.64	−1.88	390.2629, 372.2517, 358.2379, 340.2238, 98.0970	Talatizamine		++++	++
19.	420.23825	C_23_H_33_NO_6_	15.48	−0.86	402.2268, 370.1989, 293.7002, 154.1224	Giraldine F		++	
20.	452.29996	C_25_H_41_NO_6_	15.54	−1.57	420.2740, 388.2465, 356.2219, 209.1644, 154.1228, 114.0916	Chasmanine		++++	
21.	464.30038	C_26_H_41_NO_6_	16.933	−0.60	432.2740, 414.2626, 400.2474, 372.2535, 265.1608, 235.1487, 154.1225	14-Acetyltalatizamine		++++	++
22.	606.28992	C_31_H_43_NO_11_	17.26	−1.60	574.2627, 556.2545, 524.2269, 506.2188, 492.1945, 261.0641, 173.0955, 105.0341	14-Benzoyl-10-OH-mesaconine		++	
23.	544.28955	C_30_H_41_NO_8_	19.47	−0.95	512.2635, 494.2548, 480.2364, 462.2258, 390.2286, 270.0846, 105.0340	Gadenine		+	
24.	590.29490	C_31_H_43_NO_10_	19.51	−1.78	558.2616, 540.2575, 419.7593, 307.8019, 246.8854, 105.0339	14-Benzoylmesaconine		++	
25.	540.29486	C_31_H_41_NO_7_	20.01	−1.33	504.2730, 462.2614, 382.2463, 340.2256, 322.2149, 304.2042	Aconicarchamine B		+	
26.	604.31060	C_32_H_45_NO_10_	20.53	−1.68	572.2811, 554.2750, 522.2495, 490.2176, 340.3151, 105.0341	14-Benzoylaconine		++	
27.	574.30010	C_31_H_43_NO_9_	21.20	−1.65	542.2744, 510.2461, 304.5384, 198.1281, 105.0339	14-Benzoylhypaconine		+++	
28.	618.29210^[M-H]−^	C_32_H_45_NO_11_	21.21	0.31	384.9167, 351.8983, 270.7405, 190.9267	14-Benzoyl-10-OH-aconine		++	
29.	648.30023	C_33_H_45_NO_12_	21.93	−1.98	588.2775, 556.2513, 455.3509, 370.1645, 105.0340	10-OH-mesaconitine		++	
30.	558.30530	C_31_H_43_NO_8_	22.07	−1.52	526.2800, 508.2674, 232.0710182.0626, 105.0341	14-Benzoyl-doxyhypaconine		++	
31.	588.31561	C_32_H_45_NO_9_	22.32	−1.24	556.2905, 524.2639, 506.2443, 346.4250, 253.7027, 154.1226, 105.0341	14-Benzoyldeoxyaconine		+	
32.	542.31061	C_31_H_43_NO_7_	23.18	−1.15	510.2846, 492.2735, 482.2483, 460.2504, 154.1231	14-Benzoylneoline		+	
33.	632.30591	C_33_H_45_NO_11_	23.18	−0.63	572.2844, 540.2551, 522.2487, 508.2299, 354.1694, 105.0341	Mesaconitine *		+++	
34.	662.31683	C_34_H_47_NO_12_	23.39	−0.27	-	Aconifine		++	
35.	614.29553	C_33_H_43_NO_10_	24.17	−0.72	554.2743, 494.2534, 372.2162, 344.21622, 203.5583, 105.0341	2,3-didehydrohypaconitine		+	
36.	646.32135	C_34_H_47_NO_11_	24.58	−0.84	586.3002, 554.2727, 526.2797, 494.2520, 368.1843, 105.0340	Aconitine *		++	
37.	616.31079	C_33_H_45_NO_10_	24.61	−0.83	556.2903, 524.2634, 496.2750, 464.2434, 338.1741, 310.1812, 105.0341	Hypaconitine *		++++	
38.	600.31592	C_33_H_45_NO_9_	24.96	−1.32	540.2948, 508.2683, 480.2747, 476.2424, 448.2475, 354.2031, 254.4337, 105.0339	Secoyunaconitine		+	
39.	572.32117	C_32_H_45_NO_8_	24.95	−1.10	484.2688, 456.2745, 382.2002, 322.1798, 294.1857, 158.0964	14-O-Anisoylneoline		+	
40.	630.32635	C_34_H_47_NO_10_	26.07	−1.36	570.3046, 538.2788, 510.2882, 506.2528, 478.2571, 352.1898, 314.5361, 105.0341	3-Deoxyaconitine		+++	
41.	614.33173	C_34_H_47_NO_9_	27.60	−0.53	-	Chasmaconitine		++	
	Phenolic compounds								
42.	209.04474^[M-H]−^	C_10_H_10_O_5_	8.56	−3.85	165.0545, 121.0281, 103.9187, 87.9238, 59.0123	Hydroxyferulic acid		+++	++
43.	433.13394^[M-H]−^	C_18_H_24_O_12_	9.88	0.08	161.0442, 125.0230, 99.0436	Asperulosidic acid		++	
44.	433.11407^[M-H]−^	C_21_H_22_O_10_	13.87	−0.19	271.0615, 151.0024	5-Hydroxyliquiritin		++	
45.	593.15137^[M-H]−^	C_27_H_30_O_15_	15.51	0.29	473.1098, 383.9785, 353.0774	Vitexin II		+++	
46.	563.14055^[M-H]−^	C_26_H_28_O_14_	15.83	−0.51	473.1089, 443.0985, 383.0769, 253.0502, 146.9367	Vitexin I		+	
47.	417.11890^[M-H]−^	C_21_H_22_O_9_	16.74	−0.32	255.0662, 153.0182, 135.0074, 119.0488	Liquiritin *		++++	
48.	505.13339	C_24_H_24_O_12_	18.72	−0.89	257.0809, 137.0234	Malonyl liquiritin		+	
49.	505.13358	C_24_H_24_O_12_	18.99	−0.07	257.0810, 137.0234	Malonyl liquiritin		+	
50.	431.13280	C_22_H_22_O_9_	20.26	−1.97	269.0809	Ononin		++++	
51.	417.11908^[M-H]−^	C_21_H_22_O_9_	20.39	−1.01	255.0662, 153.0180, 135.0072, 119.0481	Neoliquiritin	0.75	+++	
52.	417.11900^[M-H]−^	C_21_H_22_O_9_	20.74	−2.47	255.0662, 153.01816, 135.0074, 119.0488	Isoliquiritin	1.28	++	
53.	285.07670^[M-H]−^	C_16_H_14_O_5_	21.23	−0.31	270.0536, 253.0505, 177.0182, 150.0310, 108.0203	Licochalcone B		++	
54.	255.06560	C_15_H_10_O_4_	21.30	0.10	227.0704, 199.0754, 145.0286, 137.0234	Dihydroxyflavone		++++	
55.	255.06580^[M-H]−^	C_15_H_12_O_4_	21.70	−0.36	153.0180, 135.0073, 119.0487, 91.0173	Liquiritigenin *		++++	+++
56.	295.19040	C_17_H_26_O_4_	23.34	−0.22	177.0914, 163.0755, 137.0598, 131.0493, 99.0809	6-Gingerol		++++	++
57.	269.04530^[M-H]−^	C_15_H_10_O_5_	24.34	0.38	233.1537, 181.0644	Genistein		+++	
58.	255.06586^[M-H]−^	C_15_H_12_O_4_	26.29	−0.49	153.0179, 135.0073, 119.0487, 91.0174	Isoliquiritigenin *		++++	+++
59.	269.08170	C_16_H_12_O_4_	26.58	1.04	253.0497, 237.0554, 213.0911, 118.0418, 107.0497	Formononetin *		++++	+++
60.	367.11790^[M-H]−^	C_21_H_20_O_6_	27.75	−1.99	352.0944, 309.0400, 298.0476, 283.0247	Glycycoum-arim/Licocoumarione		+++	
61.	271.09565	C_16_H_14_O_4_	28.56	1.19	254.2579, 161.0599, 137.0598, 123.04440, 100.0763	Echinatin		++	
62.	355.11835^[M-H]−^	C_20_H_20_O_6_	28.60	−1.07	328.1265, 269.11820, 269.11820, 178.9975, 125.0230	8-Dimethylallyleriodictyol/6-Dimethylallyleriodictyol		++	
63.	277.18008	C_17_H_24_O_3_	28.84	2.28	177.0912, 145.0649, 137.0598	6-Shogaol		+++	++
64.	355.15480^[M-H]−^	C_21_H_24_O_5_	29.81	−1.06	323.1284, 233.1176, 207.1017, 135.0438, 125.0230, 109.0280	Isopentadienyl glycyrrhizoflavone		++	
65.	367.11790^[M-H]−^	C_21_H_20_O_6_	29.53	−2.00	309.0400, 297.0400, 284.0325, 203.0702	Glycycoum-arim/Licocoumarione		+++	
66.	321.11262^[M-H]−^	C_20_H_18_O_4_	30.07	−1.93	306.0892, 174.9549	Licoflavone A		+	
67.	353.10290^[M-H]−^	C_20_H_18_O_6_	30.17	−1.33	339.1187, 321.1126, 295.0613, 283.0614, 270.0535	Isolicoflanonol		+++	++
68.	353.13782	C_21_H_20_O_5_	30.22	−1.59	299.0906, 297.0857, 267.0653, 199.0758, 147.0441, 135.0441	Gancaonin M		++	
69.	383.11273^[M-H]−^	C_21_H_20_O_7_	30.39	−2.33	338.2439, 247.1310, 227.0704, 207.1015, 155.0337, 140.0101	Licopyranocoumarin		+	
70.	383.14828	C_22_H_22_O_6_	30.66	−1.50	327.0859, 299.0913, 191.0704	Glycyrin		++	++
71.	355.15320	C_21_H_22_O_5_	30.94	−2.01	289.0549, 287.0553, 191.1067, 153.0548, 69.0708	Licobenzofuran/liconeolignan		+++	
72.	337.10780^[M-H]−^	C_20_H_18_O_5_	31.02	−1.07	314.0428, 282.0531	Licoflavone C		++	
73.	365.10239^[M-H]−^	C_21_H_18_O_6_	30.17	−1.63	307.0244, 295.0245, 282.0169	Isoglycyrol	4.84	++	
74.	365.10236^[M-H]−^	C_21_H_18_O_6_	31.12	−1.99	307.0242, 295.0243, 282.0167	Glycyrol	5.04	+++	
75.	333.24170	C_21_H_32_O_3_	34.17	−1.99	177.0911, 145.0649, 137.0598	10-Shogaol		++	
76.	279.23264^[M-H]−^	C_18_H_32_O_2_	38.27	1.13	261.2219, 199.8500	Linoleic acid		++	
	Saponins								
77.	879.40173^[M-H]−^ 881.41516 705.38361^[M+H-glcA]+^ 511.34122^[agl+H-H2O]+^	C_44_H_64_O_18_	24.011	−0.67 −1.38	(−) 351.0557, 193.0342, 113.0229 (+) 511.3408, 493.3279, 451.3188, 141.0183	Uralsaponin M		++	
78.	837.39105^[M-H]−^ 839.40466 469.33072^[gal+H-H2O]+^	C_42_H_62_O_17_	25.49	−0.79 −1.32	(−) 351.05603, 289.05652, 193.03430, 175.02340, 113.02294 (+) 469.3304, 487.3415, 451.3209, 141.0184	Yunganoside K2		++	
79.	837.39178^[M-H]−^ 839.40491 469.33084^[agl+H-H2O]+^	C_42_H_62_O_17_	26.07	−0.84 −1.07	(−) 351.05557, 289.05621, 193.03413, 175.02360, 113.02285 (+) 469.3304, 487.3413, 451.3198, 141.0183	Licoricesaponin G2		+	
80.	471.34613	C_30_H_46_O_4_	26.62	−1.41	453.33508, 425.34262, 317.21100, 235.16887, 189.16374	Glycyrrhetinic acid (enoxolone) *		+	
81.	821.39630^[M-H]−^ 823.40936 647.37744^[M+H-glcA]+^ 453.33554^[agl+H-H2O]+^	C_42_H_62_O_16_	26.64	1.08 −1.70	(−) 351.05573, 193.03406, 175.02338, 113.02288 (+) 453.3354, 471.3451, 435.3259	Glycyrrhizic acid *		++++	
82.	821.39612^[M-H]−^ 823.40936 647.37787^[M+H-glcA]+^ 453.33585^[agl+H-H2O]+^	C_42_H_62_O_16_	27.65	0.86 −0.47	(−) 351.05640, 193.03404, 175.02319, 113.02289 (+) 453.3354, 435.3257	Uralsaponin B or Licoricesaponine K2/H2		++	

Note: * Compounds identified by comparing with reference standards; glcA: β-D-glucuronopyronosyl; agl: aglycone; +, response area below 10^6^; ++, response area between 10^6^ and 10^7^; +++, response area between 10^7^ and 10^8^; ++++, response area above 10^8^.

**Table 3:** Metabolites of major alkaloids found in the urine and plasma samples.

ID	[M+H]^+^ (*m/z*)	Formula	t_R_ (min)	Error (ppm)	ms/ms	Composition Change	Identification	ClogP	Area	
									Urine	Plasma
M1.	410.25302	C_22_H_35_NO_6_	3.72	−1.69	392.2425, 374.2317, 360.2165, 342.2054	+O	Hydroxy karakolidine		++	
M2.	374.23212	C_22_H_31_NO_4_	7.55	−1.25	356.2212, 338.2106, 198.1122	+O	Hydroxy songorine		++	
M3.	394.25812	C_22_H_35_NO_5_	7.76	−1.85	376.2476, 358.2362, 98.0971, 58.0611	+O	Hydroxy karakoline		++	
M4.	438.28433	C_24_H_39_NO_6_	10.76	0.67	406.2588, 388.2476, 374.230, 356.2226	+O	10-Hydroxy talatizamine		++	
M5.	632.30560	C_33_H_45_NO_11_	22.61	−1.50	572.2853, 540.2590, 512.2641, 508.2310, 480.2390, 358.2004, 354.1703, 105.0341	+O	Hydroxy hypaconitine		++	
M6.	392.24268	C_22_H_33_NO_5_	8.28	−1.21	374.2315, 344.2221, 312.1962, 114.0916	-H2	Dehydrogenated karakolidine		+++	
M7.	392.24249	C_22_H_33_NO_5_	8.95	−2.15	374.2325, 344.2240	-H2	Dehydrogenated karakolidine		+++	
M8.	452.26361	C_24_H_37_NO_7_	9.85	−1.48	434.2529, 416.2419, 204.2270, 384.2155	-H2	Dehydrogenated fuziline		+++	
M9.	376.24780	C_22_H_33_NO_4_	10.38	−1.82	358.2373, 98.0969	-H2	Dehydrogenated karakoline		++	
M10.	376.24756	C_22_H_33_NO_4_	10.96	−1.15	358.2375, 234.0137, 98.0970	-H2	Dehydrogenated karakoline		+++	
M11.	436.26895	C_24_H_37_NO_6_	10.24	−0.95	418.2581, 400.2475, 386.2315, 358.2355, 340.2265	-H2	Dehydrogenated neoline		+++	+
M12.	436.26907	C_24_H_37_NO_6_	10.64	−0.67	418.2585, 400.2473, 386.2303, 358.2383	-H2	Dehydrogenated neoline		+++	
M13.	420.27393	C_24_H_37_NO_5_	13.60	−1.33	388.2477, 370.2375, 98.0972	-H2	14-Dehydrogenated talatizamine		+++	
M14.	364.24744	C_21_H_33_NO_4_	7.39	−2.20	346.2374, 328.2268	-CH2	Demethyl karakoline		+++	+
M15.	364.24740	C_21_H_33_NO_4_	7.86	−2.20	346.2370, 328.2266	-CH2	Demethyl karakoline		++++	+
M16.	424.26892	C_23_H_37_NO_6_	10.68	−1.05	406.2583, 374.2327, 356.2211, 342.2069, 154.1226	-CH2	Demethyl neoline		+++	+
M17.	424.26890	C_23_H_37_NO_6_	11.08	−0.98	406.2581, 374.2317, 356.2222, 342.2076, 154.1228	-CH2	Demethyl neoline		++++	
M18.	408.27386	C_23_H_37_NO_5_	9.87	−1.44	376.2475, 358.2365, 326.2136	-CH2	18-*o*-Demethyl talatizamine	−0.78	++++	++
M19.	408.27393	C_23_H_37_NO_5_	11.17	−1.29	376.2478, 358.2373, 326.2129	-CH2	16-*o*-Demethyl talatizamine	−0.73	++++	++
M20.	602.29486	C_32_H_43_NO_10_	21.31	−1.70	542.2742, 510.2477, 482.2540, 478.2212, 324.1592, 105.0339	-CH2	Demethyl hypaconitine		++++	+
M21.	618.28992	C_32_H_43_NO_11_	22.17	−1.22	558.2684, 526.2423, 508.2394, 354.1695, 105.0341	-CH2	Demethyl mesaconitine		++	
M22.	330.20581	C_20_H_27_NO_3_	8.19	−1.70	312.1954	-C2H4	Deethyl songorine		+++	
M23.	350.23181	C_20_H_31_NO_4_	9.05	−2.21	332.2215, 314.2106, 300.1958, 234.9901, 158.9743	-C2H4	Deethyl karakoline		+++	
M24.	410.25314	C_22_H_35_NO_6_	10.97	−1.40	392.2423, 378.2271, 360.2163, 328.1906	-C2H4	Deethyl neoline		++	
M25.	408.27374	C_23_H_37_NO_5_	8.96	−1.74	390.2631, 372.2537, 358.2369	-CH2O	Demethyl-deoxy neoline		++++	++
M26.	392.27896	C_23_H_37_NO_4_	13.38	−1.46	360.2527, 342.2436, 328.2265	-CH2O	16-O-Demethyl-14-deoxy Talatizamine		+++	
M27.	602.29529	C_32_H_43_NO_10_	23.87	−1.20	542.2773, 510.2486, 478.2222, 324.1592, 105.0341	-CH2O	Demethyl-deoxy mesaconitine		+++	
M28.	360.25272	C_22_H_33_NO_3_	9.38	−1.68	342.2422, 324.2325, 121.0651	-H2O	Dehydrated karakoline		+++	+
M29.	360.25250	C_22_H_33_NO_3_	9.92	−2.11	342.2422, 324.2307	-H2O	Dehydrated karakoline		++++	+
M30.	614.2517	C_33_H_43_NO_10_	24.17	−1.57	544.2743, 522.2518, 494.2534, 372.2162, 344.2215	-H2O	Dehydrated mesaconitine		++	
M31.	380.24277	C_22_H_33_NO_5_	8.81	−1.01	362.2316, 344.2046, 330.2065	-CH2+O	Demethyl-hydroxy karakoline		++	
M32.	438.24811	C_23_H_35_NO_7_	12.09	−1.06	420.2374, 402.2265, 392.2440, 374.2317	-CH2-H2	Dehydrogenated-demethyl fuziline		++	+
M33.	438.24890	C_23_H_35_NO_7_	12.21	0.49	420.2367, 402.2283, 392.2442, 374.2323	-CH2-H2	Dehydrogenated-demethyl fuziline		++	+
M34.	362.23172	C_21_H_31_NO_4_	8.15	−0.45	344.2223, 185.0710	-CH2-H2	Dehydrogenated-demethyl karakoline		+++	
M35.	422.25327	C_23_H_35_NO_6_	10.99	−1.07	390.2268, 406.2597, 390.2268, 374.2324	-CH2-H2	Dehydrogenated-demethyl neoline		+++	+
M36.	406.25839	C_23_H_35_NO_5_	10.70	−1.01	388.2477, 370.2368, 328.2266	-CH2-H2	14-Dehydrogenated-16-O-demethyl talatizamine		+++	
M37.	346.20090	C_20_H_27_NO_4_	7.79	−1.12	328.1904, 296.1645, 268.1701, 251.1437	-C2H4+O	N-Deethyl-hydroxy songorine		++	+
M38.	346.20071	C_20_H_27_NO_4_	8.49	−1.65	328.1903, 296.1650, 268.1699, 251.1429	-C2H4+O	N-Deethyl-hydroxy songorine		+++	
M39.	378.26337	C_22_H_35_NO_4_	10.98	−1.43	346.2371, 328.2279	-C2H4-O	N-Deethyl-14-deoxy talatizamine	0.88	+++	+
M40.	378.26334	C_22_H_35_NO_4_	11.23	−1.45	346.2371, 328.2267	-C2H4-O	N-Deethyl-8-deoxy talatizamine	1.15	+++	

Note: +, response area below 10^6^; ++, response area between 10^6^ and 10^7^; +++, response area between 10^7^ and 10^8^; ++++, response area above 10^8^.

**Table 4:** Metabolites of phenolic compounds found in the urine and plasma samples.

ID	[M+H]^+^ (*m/z*)	Formula	t_R_ (min)	Error (ppm)	ms/ms	Composition Change	Identification	Area	
								Urine Plasma	
M41.	259.09701	C_15_H_14_O_4_	17.57	2.53	153.0548, 135.0441, 107.0496	+H2	Hydrogenated liquiritigenin	++++	++
M42.	259.09689	C_15_H_14_O_4_	20.78	1.83	153.0549, 107.0497	+H2	Hydrogenated isoliquiritigenin	++++	+++
M43.	297.20602	C_17_H_28_O_4_	22.11	−0.23	177.0912, 163.0755, 137.0598, 131.0494	+H2	Hydrogenated 6-gingerol	++++	++
M44.	269.08170^[M-H]−^	C_16_H_14_O_4_	26.82	−0.03	254.0582, 153.0178, 135.0073, 91.0173	+H2	Hydrogenated formononetin	++++	++
M45.	367.11792^[M-H]−^	C_21_H_20_O_6_	29.54	−2.01	352.0936, 309.0400, 310.0434, 284.0325	+H2	Hydrogenated glycyrol	+++	+
M46.	355.15311	C_21_H_22_O_5_	30.71	−2.57	337.1065, 299.0912, 189.0911, 177.0546, 151.0393	+H2	Hydrogenated gancaonin M	++	
M47.	309.20578	C_18_H_28_O_4_	26.17	−0.70	163.0756, 137.0599, 131.0494	+CH2	Methyl 6-gingerol	++	
M48.	309.20572	C_18_H_28_O_4_	26.80	−0.92	179.0704, 150.068, 137.0598, 83.0864	+CH2	Methyl 6-gingerol	++	
M49.	285.07587	C_16_H_12_O_5_	18.49	0.51	270.0525, 253.0499, 299.0866, 225.0546, 123.0443	+O	Hydroxy formononetin	+++	
M50.	273.07593	C_15_H_12_O_5_	19.06	0.72	255.066, 179.0339, 153.0184, 147.0442, 123.044, 119.0496	+O	Hydroxy liquiritigenin/isoliquiritigenin	+++	++
M51.	369.13266	C_21_H_20_O_6_	29.18	−1.71	351.1222, 229.0860, 193.0497, 165.0548, 151.0389	+O	Hydroxy gancaonin M	+++	+
M52.	313.20038	C_17_H_28_O_5_	15.07	−0.96	203.1066, 163.0754, 137.0598	+H2O	Hydrated 6-gingerol	+++	
M53.	273.07629^[M-H]−^	C_15_H_14_O_5_	20.08	−1.36	255.0661, 167.0337, 109.0279	+H2O	Hydrated liquiritigenin	+++	
M54.	273.07660^[M-H]−^	C_15_H_14_O_5_	23.05	−0.68	255.0655, 151.0387, 135.0072, 109.0280	+H2O	Hydrated isoliquiritigenin	+++	
M55.	293.17447	C_17_H_24_O_4_	16.57	−0.91	163.0756, 137.0598, 99.0811	-H2	Dehydrogenated 6-gingerol	++	+
M56.	255.06552	C_15_H_10_O_4_	18.49	0.41	227.0703, 199.0756, 137.0234	-H2	Dehydrogenated liquiritigenin	+++	+
M57.	255.06550	C_15_H_10_O_4_	21.29	−0.16	227.0699, 199.0755, 137.0234	-H2	Dehydrogenated isoliquiritigenin	++++	+
M58.	307.15466^[M-H]−^	C_17_H_24_O_5_	26.17	−1.41	275.1288, 171.1014, 153.0907, 121.0280, 111.0799	-H2+O	Dehydrogenated-hydroxy 6-gingerol	++	
M59.	277.18039	C_17_H_24_O_3_	28.85	2.07	189.0914, 177.09123, 145.05493, 137.0597	-H2-O	Dehydrated 6-gingerol	++++	++
M60.	285.07648^[M-H]−^	C_16_H_14_O_5_	22.03	−0.35	270.0533, 153.0180, 149.0594, 135.0073, 134.0358, 91.0174	+CH2+O	Methyl-hydroxy liquiritigenin	+++	+
M61.	285.07645^[M-H]−^	C_16_H_14_O_5_	26.88	−1.06	270.0535, 153.0180, 149.0595, 135.0073, 91.0174	+CH2+O	Methyl-hydroxy isoliquiritigenin	++	
M62.	299.09170	C_17_H_14_O_5_	26.99	0.69	284.0680, 243.1061, 166.0268	+CH2+O	Methyl-hydroxy formononetin	+++	+
M63.	335.02261^[M-H]−^	C_15_H_12_O_7_S	18.62	−1.33	255.0661, 199.0064, 135.0073, 119.0487	+SO3	Liquiritigenin sulfate	++++	+++
M64.	335.02271^[M-H]−^	C_15_H_12_O_7_S	23.32	−0.94	255.0663, 199.0055, 135.0073, 119.0486	+SO3	Isoliquiritigenin sulfate	++++	++++
M65.	347.02263^[M-H]−^	C_16_H_12_O_7_S	23.56	−1.41	267.0664, 252.0427	+SO3	Formononetin sulfate	++++	+++

Note: +, response area below 10^6^; ++, response area between 10^6^ and 10^7^; +++, response area between 10^7^ and 10^8^; ++++, response area above 10^8^.

## Data Availability

The data presented in this study are available on request from the corresponding author. The data are not publicly available due to confidentiality.

## Supplementary Materials

The following supporting information can be downloaded at https://www.mdpi.com/article/10.3390/metabo14060333/s1, Figure S1: Workflow for the identification of prototype components, Figure S2: Workflow for the identification of metabolites, Figure S3: MS/MS spectra of major prototype compounds in the urine samples. Table S1: Compound information of in-house database, Table S2: Parameters of data processing by Compound Discoverer software.
