# Supplementary material for: Metabolite Profiling Analysis of the Tongmai Sini Decoction in Rats after Oral Administration through UHPLC-Q-Exactive-MS/MS
Source: Metabolites. 2024 Jun 14;14(6):333. doi: 10.3390/metabo14060333 (PMC11205536; doi:10.3390/metabo14060333)
Supplement: Supplementary file 1 [file metabolites-14-00333-s001.zip › Table S1 Compound information of in-house database.pdf]

Table S1. Compound information of in-house database.

No.	Name	Formula
1.	karakolidine	C22H35O5N
2.	9-Hydroxysenbusine A	C23H37O7N
3.	mesaconine	C24H39O8N
4.	Senbusine A/senbusine B	C23H37O6N
5.	Isotalatizidine	C23H37O5N
6.	Senbusine B/Senbusine A	C24H37O6N
7.	Talatizidine	C23H37O5N
8.	Karakoline	C22H35O4N
9.	Aconine	C25H41O9N
10.	15-Hydroxyneoline	C24H39O7N
11.	Napelline	C22H33O3N
12.	Songorine	C22H31O3N
13.	Unknown	C24H37O7N
14.	Hypaconine	C24H39O8N
15.	Fuziline	C24H39O7N
16.	Neoline	C24H39O6N
17.	Unknown	C30H49O11N
18.	Bikhaconine	C25H41O7N
19.	14-Acetylkarakoline	C24H37O5N
20.	Deoxyaconine	C25H41O8N
21.	unknown	C24H37O7N
22.	N-methyl-laurotetanine	C20H23O4N
23.	Talatizamine	C24H39O5N
24.	Chasmanine	C25H41O6N
25.	14-Dehydro-19-oxodelcosine	C24H35O8N
26.	Delsoline	C25H41O7N
27.	7-deoxylycoctonine	C25H41O6N
28.	Giraldine F	C23H33O6N
29.	N-deethyl-14-benzoylaconine	C30H41O10N
30.	Unknown	C30H39O10N
31.	14-Acetyltalatizamine	C26H41O6N
32.	14-Benzoyl-10-OH-mesaconine	C31H43O11N
33.	Unknown	C30H39O10N
34.	unknown	C25H35O6N
35.	Hemsleyanine E	C30H41O9N
36.	Unknown	C30H41O10N
37.	14-Benzoyl-10-OH-aconine	C32H45O11N
38.	Isomer of 14-Benzoylhypaconine	C31H43O9N
39.	Unknown	C30H41O10N
40.	Gadenine	C30H41O8N
41.	14-Benzoylmesaconine	C31H43O10N

42.	N-demethyl-14-benzoylhapoconine	C30H41O9N
43.	Aconicarchamine B	C31H41O7N
44.	Isomer of 14-Benzoylhypaconine	C31H43O9N
45.	14-Benzoylaconine	C32H45O10N
46.	Isomer of 14-Benzoylhypaconine	C31H43O9N
47.	10-OH-15-deoxymesaconitine	C33H45O11N
48.	14-Benzoylhypaconine	C31H43O9N
49.	Unknown	C29H41O7N
50.	Secojesaconitine	C33H45O10N
51.	Karaconitine	C34H47O11N
52.	Dehydrated benzoylmesaconine	C31H41O9N
53.	14-Benzoyl-doxyhypaconine	C31H43O8N
54.	10-OH-mesaconitine	C33H45O12N
55.	14-Benzoyldeoxyaconine	C32H45O9N
56.	Unknown	C30H41O8N
57.	Unknown	C29H41O6N
58.	14-Benzoylneoline	C31H43O7N
59.	Indaconitine	C34H47O10N
60.	N-Demethyl-mesacontine	C32H43O11N
61.	10-OH-hepaconitne	C33H45O11N
62.	Isomer of 10-OH-hepaconitne	C33H45O11N
63.	Unknown	C31H43O7N
64.	Secoyunaconitine	C33H45O9N
65.	14-Benzoyl-16-ketoneoline	C32H43O9N
66.	8-Methoxyl-14-benzoylaconine	C33H47O10N
67.	Mesaconitine	C33H45O11N
68.	Dehydrated benzoylhypaconine	C31H41O8N
69.	10-OH-15-deoxyaconitine	C34H47O11N
70.	Aconifine	C34H47O12N
71.	Dehydrated 14-benzoyldeoxyaconine	C32H43O8N
72.	14-Benzoyl-methoxy-hypaconine	C32H45O9N
73.	Dehydrated benzoylhypaconine	C31H41O8N
74.	N-demethylhypaconitine	C32H43O10N
75.	8-O-Et-14-Benzoylmesaconine	C33H47O10N
76.	Dehydro-mesaconitine	C33H43O10N
77.	8-Acetyl-14-benzoylneoline	C33H45O8N
78.	14-O-anisoylneoline	C32H45O8N
79.	8-O-cinnamoylneoline	C33H45O7N
80.	Hypaconitine	C33H45O10N
81.	Dehydrated benzoylhypaconine	C32H45O7N
82.	Aconitine	C34H47O11N
83.	14-O-veratroylneoline	C33H47O9N
84.	Isodelphinine	C33H45O9N

85.	14-O-cinnamoylneoline	C33H45O7N
86.	Veratroylbikhaconine	C34H49O10N
87.	Unknown	C32H45O8N
88.	Delphinine	C33H45O9N
89.	3-Dehydroaconitine	C34H45O10N
90.	Acotoxinine	C33H47O9N
91.	Unknown	C39H41O11N
92.	Chasmaconitine	C34H47O9N
93.	3-Deoxyaconitine	C34H47O10N
94.	3, 13-Deoxyaconitine	C34H47O9N
95.	Unknown	C39H41O9N
96.	8-Nonanedioic acid-benzoyhepaconine	C40H57O12N
97.	Unknown	C43H47O12N
98.	8-Hydroxyl linoleic acid-benzoylhypaconine	C49H73O11N
99.	8-Stearic acid-benzoyldeoxyaconine	C49H75O11N
100.	8-Linolenic acid-benzoylmesaconine	C49H71O11N
101.	8-Lnoleic acid-10-OH-benzoylmesaconitine	C49H73O12N
102.	8-Linolenic acid-benzoylaconine	C50H73O11N
103.	8-Palmitoleic acid-benzoylmesaconine	C47H71O11N
104.	8-Linolenic acid-benzoylhepaconine	C49H71O10N
105.	8-Linoeic acid-benzoyl-10-OH-aconine	C50H75O12N
106.	8-Linoleic acid-benzoylmesaconine	C49H73O11N
107.	8-Pentadecanoic acid-benzoylmesaconine	C46H71O11N
108.	8-Palmitic acid-10-OH-benzoylmesaconitine	C47H73O12N
109.	8-Palmitoleic acid-benzoylhepaconine	C48H75O9N
110.	8-Oleic acid-10-OH-benzoylmesaconitine	C49H75O12N
111.	8-Linoeic acid-benzoylaconine	C50H75O11N
112.	8-Linoeic acid-benzoylaconine	C50H75O11N
113.	8-Linoeic acid-benzoylhepaconine	C49H73O10N
114.	8-Pentadecanoic acid-benzoylhepaconine	C46H71O10N
115.	8-Palmitic acid-benzoylmesaconine	C47H73O11N
116.	8-Oleic acid-benzoylmesaconine	C49H75O11N
117.	8-Linoeic acid-benzoyldeoxyaconine	C50H75O10N
118.	8-Palmitic acid-benzoylaconine	C48H75O11N
119.	8-Oleic acid-benzoylaconine	C50H77O11N
120.	8-Palmitic acid-benzoylhepaconine	C47H73O10N
121.	8-Palmitic acid-benzoylhepaconine	C47H73O10N
122.	8-Linoeic acid-benzoyl-3,13-deoxyaconine	C50H75O9N
123.	8-Oleic acid-benzoylhepaconine	C49H75O10N
124.	8-Palmitic acid-benzoyldeoxyaconine	C48H75O10N
125.	8-Oleic acid-benzoyldeoxyaconine	C50H77O10N
126.	8-Stearic acid-benzoylmesaconine	C49H77O11N

127.	8-Palmitic acid-benzoyl-3,13-deoxyaconine	C48H75O9N
128.	8-Stearic acid-benzoylhepaconine	C49H77O10N
129.	Isoleucine	C6H13NO2
130.	Phenylalanine	C9H11NO2
131.	(Z)-citral	C10H16O
132.	Galanganol C	C27H28O5
133.	Zingerone	C11H14O3
134.	Dihydrocurcumin	C21H22O6
135.	Tetrahydrocurcumin	C21H24O6
136.	(E)-7-(3,4-dihydroxyphenyl)-1-(4-hydroxy-3-methoxyphenyl)hept-2-en-1-one	C20H22O5
137.	1,5-epoxy-3-hydroxy-1-(3,4-dihydroxy-5-methoxyphenyl)-7-(4-hydroxy-3-methoxyphenyl)heptane	C21H26O7
138.	5-hydroxy-1-(4-hydroxy-3-methoxyphenyl)-7-(4-hydroxyphenyl)-3-heptanone	C20H24O5
139.	Methyl diacetoxy-[4]-gingerdiolC20H30O6	C20H30O6
140.	3,5-dihydroxy-1-(4-hydroxy-3,5-dimethoxyphenyl)-7-(4-hydroxy-3-methoxyphenyl)heptane	C22H30O7
141.	[4]-Shogaol	C15H20O3
142.	1,5-epoxy-3-hydroxy-1-(4-hydroxy-3,5-dimethoxyphenyl)-7-(4-hydroxy-4-methoxyphenyl)heptane	C22H28O7
143.	Gingerenone B	C22H26O6
144.	3-acetoxy-5-hydroxy-1-(4-hydroxyphenyl)-7-(3,4-dihydroxyphenyl)heptane	C21H26O6
145.	Dihydro-[6]-paradol	C17H28O3
146.	3,5-diacetoxy-1,7-bis(3,4-dihydroxy-5-methoxyphenyl)heptane	C25H32O10
147.	3-acetoxy-5-hydroxy-1-(3,4-dihydroxy-5-methoxyphenyl)-7-(4-hydroxy-3-methoxyphenyl)heptane	C23H30O8
148.	3,5-diacetoxy-1-(3,4-dihydroxyphenyl)-7(4-hydroxyphenyl)heptane	C23H28O7
149.	3-acetoxy-5-hydroxy-1-(3,4-dihydroxy-5-methoxyphenyl)heptane	C24H32O8
150.	3,5-diacetoxy-1-(3,4-dihydroxyphenyl)-7-(3,4-dihydroxy-5-methoxyphenyl)heptane	C24H30O9
151.	3-acetoxy-5-hydroxy-1-(3,4-dihydroxy-5-methoxyphenyl)heptane	C24H32O8
152.	3,5-diacetoxy-1-(3,4-dihydroxyphenyl)-7-(3,4-dihydroxy-5-methoxyphenyl)heptane	C24H30O9
153.	Dehydro-[6]-gingerdione	C17H22O4
154.	Trihydroxy octadecenoic acid	C18H34O5
155.	Curcumadiol	C15H26O2
156.	3,5-diacetoxy-1-(3,4-dihydroxy-5-methoxyphenyl)-7-(4-hydroxy-3,5-dimethoxyphenyl)heptane	C26H34O10
157.	[6]-Paradol	C17H26O3
158.	3,5-diacetoxy-1-(4-hydroxy-3-methoxyphenyl)-7-(3, 4-dihydroxy-5-methoxyphenyl)heptane	C25H32O9
159.	[6]-Gingerol	C17H26O4
160.	6-hydroxy-[6]-shogaol	C17H24O4
161.	3,5-diacetoxy-1-(4-hydroxy-3,5-dimethoxyphenyl)-7-(4-hydroxy-3-methoxyphenyl)heptane	C26H34O9

162.	3,5-diacetoxy-1-(4-hydroxy-3-methoxyphenyl)-7-(4-hydroxyphenyl)heptane	C24H30O7
163.	1,7bis-(4-hydroxy-3-methoxyphenyl)-5-methoxy-3-heptanone	C22H28O6
164.	3,5-diacetoxy-1,7-bis(4-hydroxy-3-methoxyphenyl)heptane	C25H32O8
165.	Palmitic acid	C16H32O2
166.	Diacetoxy-[4]-gingerdiol	C19H28O6
167.	Acetoxy-[6]-gingerol	C19H28O5
168.	[6]-shogaol	C17H24O3
169.	Diacetoxy-[6]-gingerdiol	C21H32O6
170.	Dehydro-[8]-gingerol	C19H28O4
171.	Methyl,diacetoxy-[6]-gingerdiol	C22H34O6
172.	Dehydro-10-gingerdione	C21H30O4
173.	[6]-gingerdiol	C17H28O4
174.	[10]-shogaol	C21H32O3
175.	Acetoxy-[10]-gingerol	C23H36O5
176.	6-hydroxy-[10]-shogaol	C21H32O4
177.	Oleamide	C18H35NO
178.	Dehydro-[12]-gingerdione	C23H34O4
