# Supplementary material for: Metabolite Profiling Analysis of the Tongmai Sini Decoction in Rats after Oral Administration through UHPLC-Q-Exactive-MS/MS
Source: Metabolites. 2024 Jun 14;14(6):333. doi: 10.3390/metabo14060333 (PMC11205536; doi:10.3390/metabo14060333)
Supplement: Supplementary file 1 [file metabolites-14-00333-s001.zip › Table S2 Parameters of data processing by Compound Discoverer software.pdf]

Table S2. Parameters of data processing by Compound Discoverer software.

Parameters of 'Generate Expected Compounds'	1.Compounds Selection	Compounds	shown in Table 2
	2.Dealkylation	Apply Dealkylation	True
		Apply Dealkylation	True
		Max.Steps	1
		Min.Mass[Da]	200
	3.Transformations	Phase I	deethylation; dehydration; Glucoside hydrolysis; hydration; Oxidation; Reduction; deoxidize; dehydro; demethylation
		Phase II	Acetylation; Arginine Conjugation; Cystenine Conjugation; Glucoside; Glucuronide; Glutamine Conjugation; GSH Conjugation; Methylation; sulfation
		Max.#Phase II	2
		Max.#All Steps	3
	4.Ionization	Ions:	[M+Cl]-1; [M+FA-H]-1; [M+H]+1; [M-H+K]-1; [M-H+HAc]-1; [M-H+TFA]-1; [M-H-H2O]-1 ; [M-H]-1
Parameters of 'Filter By Mass Defect'	1.General Settings	Filter Direction	Keep
		Mass Defect Type	Standard Mass Defect
	2. Tolerances	Mass Tolerance	50Da
		Mass Defect Tolerance	0.05
	3.Custom Compositions	Ions	[M+H]+1; [M-H]-1
Parameters of 'Detect Compounds'	1.General Settings	Mass Tolerance	5ppm
		Intensity Tolerance%	5
		S/N Threshold	3
		Min. Peak Intensity	100000
		Ions	[M+H]+1; [M-H]-1
		Min.Element Counts	CH
		Max.Element Counts	C90H90N3O90S3
