# Supplementary figures and images for: Metabolite Profiling Analysis of the Tongmai Sini Decoction in Rats after Oral Administration through UHPLC-Q-Exactive-MS/MS
Source: Metabolites. 2024 Jun 14;14(6):333. doi: 10.3390/metabo14060333 (PMC11205536; doi:10.3390/metabo14060333)

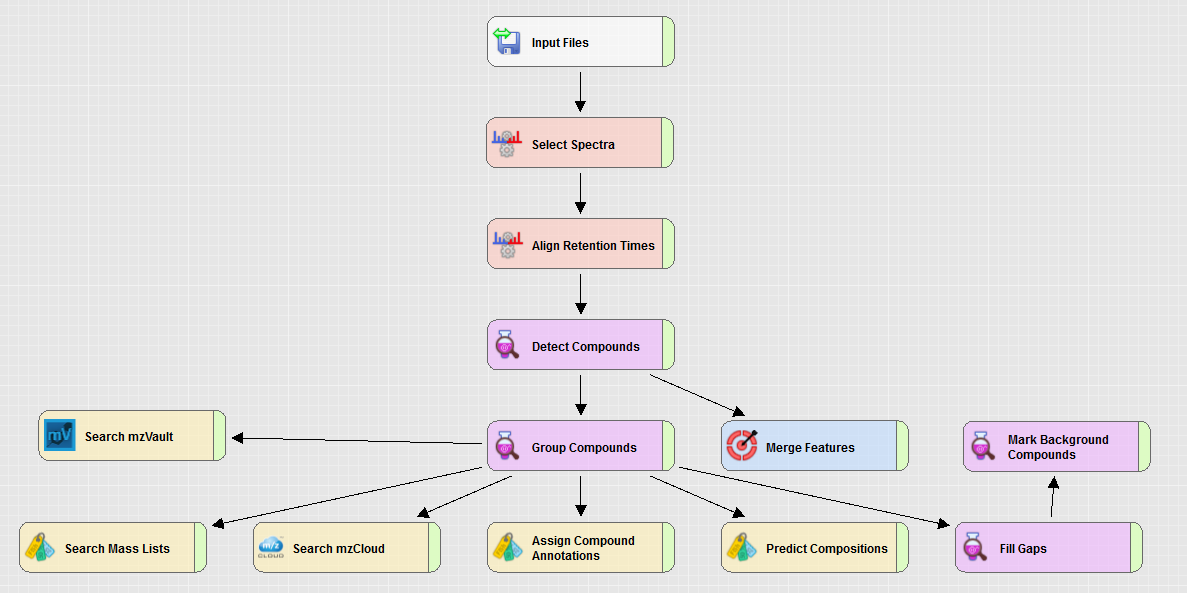

Supplement: Supplementary file 1 [file metabolites-14-00333-s001.zip › Figure S1 workflow for the identification of prototype components.tif]

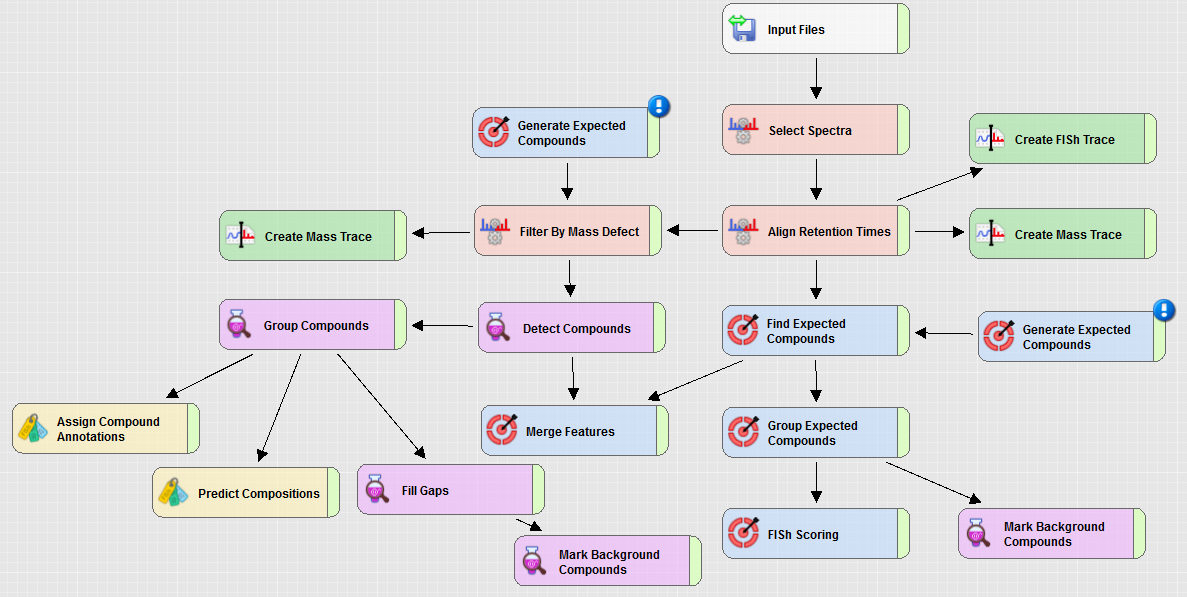

Supplement: Supplementary file 1 [file metabolites-14-00333-s001.zip › Figure S2 workflow for the identification of metabolites.tif]

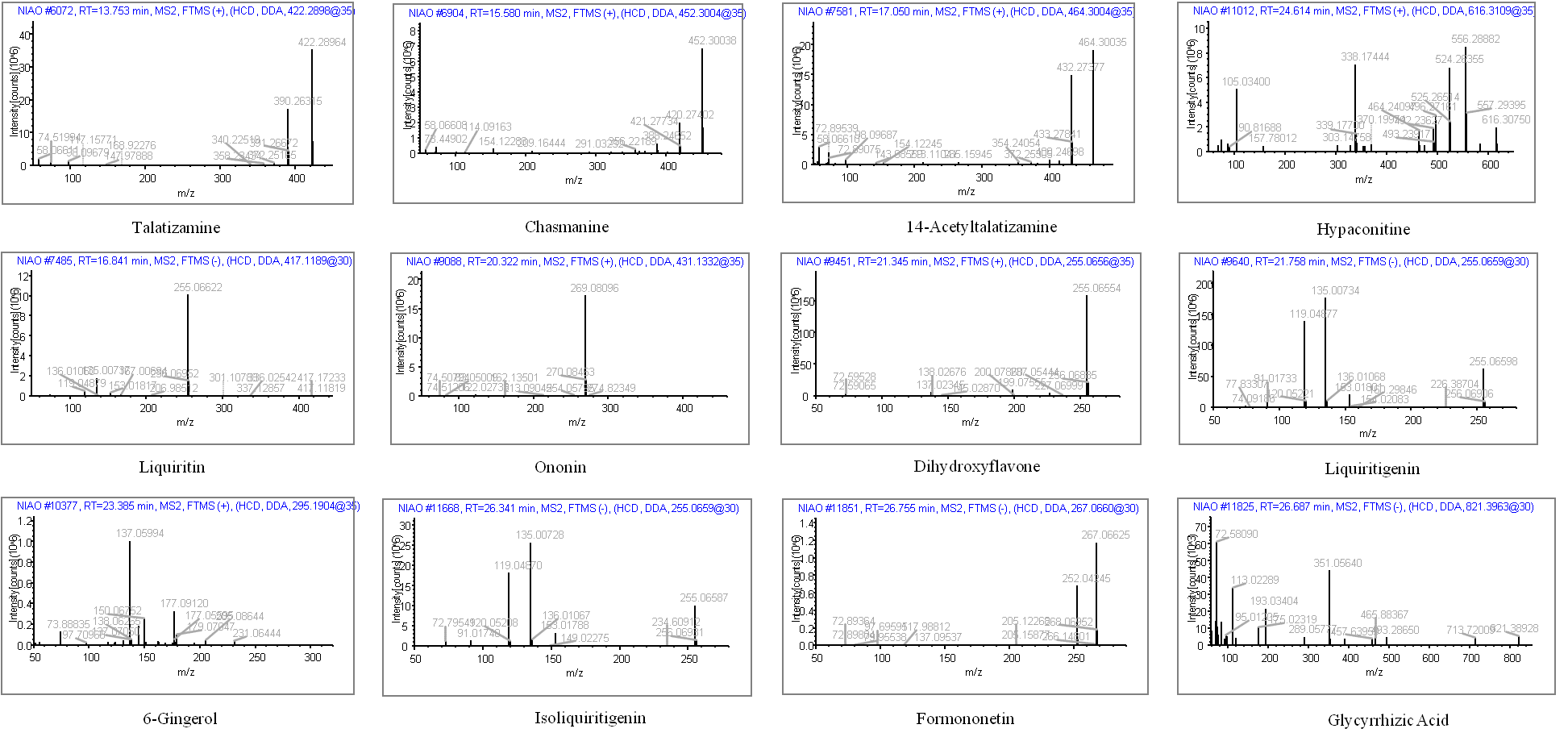

Supplement: Supplementary file 1 [file metabolites-14-00333-s001.zip › Figure S3 MSMS spectra of prototype compounds in the urine samples.tif]
